# Artificial Intelligence as a Catalyst for Cell-Based Therapies in Retinal Disease

**DOI:** 10.3390/cells15201887

**Published:** 2026-10-16

**Authors:** Girish K. Srivastava, Priyank Shukla, Sara Crespo Millas, Yogeswaran Lokanathan

**Affiliations:** 1 Departamento de Cirugía, Oftalmología, Otorrinolaringología y Fisioterapia, Facultad de Medicina, Universidad de Valladolid, 47003 Valladolid, Spain; 2 Instituto Universitario de Oftalmobiología Aplicada (IOBA), Universidad de Valladolid, 47011 Valladolid, Spain; 3 Department of Tissue Engineering & Regenerative Medicine (DTERM), Faculty of Medicine, Universiti Kebangsaan Malaysia, Kuala Lumpur 56000, Malaysia; lyoges@ukm.edu.my; 4 Personalised Medicine Centre, School of Medicine, Ulster University, C-TRIC Building, Altnagelvin Area Hospital, Glenshane Road, Londonderry BT47 6SB, UK; p.shukla@ulster.ac.uk; 5 Hospital Clínico Universitario de Valladolid, 47003 Valladolid, Spain; screspomillas@gmail.com; 6 Advance Bioactive Materials-Cells UKM Research Group, Universiti Kebangsaan Malaysia, Bangi 43600, Malaysia

**Keywords:** artificial intelligence, machine learning, deep learning, retinal disease, cell-based therapy, induced pluripotent stem cells, CRISPR-Cas9, age-related macular degeneration, retinitis pigmentosa, retinal organoids

## Abstract

Retinal degenerative diseases, including age-related macular degeneration (AMD), retinitis pigmentosa (RP), and Stargardt disease, represent leading causes of irreversible vision loss worldwide, collectively affecting hundreds of millions of people. Cell-based therapies, particularly those leveraging induced pluripotent stem cells (iPSCs), retinal pigment epithelium (RPE) transplantation, and CRISPR-Cas9 gene editing, have emerged as promising treatment modalities that may preserve, replace, or partially restore retinal function. However, these approaches face significant challenges in patient stratification, manufacturing consistency, surgical precision, and long-term outcome monitoring. Artificial intelligence (AI), encompassing machine learning (ML), deep learning (DL), and computer vision, is increasingly being integrated across the cell therapy pipeline to address these limitations. This review comprehensively explores the role of AI in enhancing cell-based retinal therapies, spanning disease diagnosis and patient selection, stem cell quality control and differentiation optimization, CRISPR off-target detection, retinal organoid characterization, scalable manufacturing, and post-transplantation monitoring through multimodal retinal imaging. Throughout, we distinguish direct retinal cell-therapy evidence from retinal-adjacent, cross-domain, and conceptual evidence. We also propose an AI-powered end-to-end pipeline that connects patient profiling through gene editing, cell production, and transplantation to longitudinal outcomes tracking (a forward-looking framework that needs to be clinically validated in future). While AI offers powerful tools to enhance efficacy, reproducibility, and personalization, it does not replace the clinical and biological foundations of cell therapy. Critical challenges remain, including data scarcity, algorithmic interpretability, regulatory compliance, and clinical validation. This review concludes that the strategic integration of AI has plausible potential to become supportive infrastructure for scalable and reproducible cell-based treatments for retinal diseases, although its necessity has not been demonstrated and prospective clinical validation is still required, and biological insight and rigorous empirical verification remain fundamental.

## 1. Introduction

Retinal degenerative diseases, including AMD, RP, and Stargardt disease, are leading causes of irreversible vision loss, collectively affecting over 300 million individuals worldwide [1,2,3,4]. Because the neural retina lacks meaningful regenerative capacity, current pharmacological and gene therapy approaches remain insufficient once photoreceptors or RPE cells are permanently lost, irrespective of the underlying genetic etiology. Cell-based therapies offer a fundamentally different therapeutic logic: rather than modifying gene function in surviving cells, they aim to replace lost retinal tissue through transplantation of exogenous cell populations. Early-phase clinical trials using human embryonic stem cell (hESC)-derived RPE provided early evidence of safety and exploratory signals of efficacy in AMD and Stargardt disease [5,6], while the advent of iPSC reprogramming technology [7,8] and CRISPR-Cas9 gene editing [9] has since made patient-specific, genetically corrected grafts conceivable, which may reduce, although not completely eliminate, the immunological limitations of allogeneic products. However, the clinical translation of these combined approaches remains constrained by challenges of manufacturing scale and reproducibility, patient stratification, surgical precision, and long-term outcome surveillance, bottlenecks that have proved resistant to conventional solutions [10,11,12].

Artificial intelligence (AI), broadly encompassing machine learning (ML), deep learning (DL), and computer vision, and implemented through convolutional neural networks (CNNs), transformer architectures, reinforcement learning, and generative models, has emerged as a promising technology to address these translational bottlenecks. DL systems trained on large-scale retinal imaging datasets have demonstrated diagnostic accuracy rivaling expert clinicians for AMD, diabetic retinopathy, and glaucoma [13,14,15], while ML approaches have been applied to optimize iPSC colony selection [16], predict CRISPR off-target editing landscapes [17] and support monitoring and control of biological manufacturing processes in real time [18]. The convergence of these capabilities across the cell therapy pipeline suggests that AI may have a role beyond diagnostics in supporting precision regenerative medicine workflows; this remains to be demonstrated. Despite these parallel advances, the intersection of AI and cell-based retinal therapy has not been comprehensively synthesized. This review examines the evidence for AI as a catalyst across all stages of the cell-based retinal therapy pipeline, from patient stratification and gene editing safety assessment through manufacturing quality control, intraoperative guidance, and post-transplantation monitoring, and proposes a conceptual framework for their integrated deployment. Throughout, we explicitly distinguish (i) direct retinal cell-therapy evidence, (ii) retinal-adjacent evidence, (iii) cross-domain proof-of-concept evidence, and (iv) conceptual or proposed applications. By mapping available evidence against translational readiness, we aim to provide a practical and forward-looking synthesis for researchers, clinicians, and developers working at this emerging interdisciplinary frontier.

### 1.1. Burden of Retinal Diseases

Retinal diseases, spanning inherited retinal dystrophies such as RP and Stargardt disease, and acquired degenerative or vascular conditions including AMD, diabetic macular edema (DME), retinal vein occlusion (RVO), and diabetic retinopathy, collectively represent the leading causes of irreversible vision loss worldwide, yet share a critical unmet need. No curative therapy currently exists for any of these conditions once significant photoreceptor or RPE cell loss has occurred [1,3,4,19,20,21]. AMD, in contrast to RP and Stargardt disease, is a multifactorial, acquired degenerative disease with genetic and environmental risk factors and is not classified as an inherited retinal dystrophy.

AMD alone affects approximately 200 million people globally, with dry AMD responsible for ~85–90% of cases [1]. The Food and Drug Administration (FDA) approved the complement inhibitors Pegcetacoplan (2023) and Avacincaptad pegol (August 2023) for geographic atrophy (GA) secondary to AMD. These therapies slow the progression of GA lesions, although they do not regenerate retinal tissue or restore lost vision. The phase 3 OAKS and DERBY trials demonstrated that pegcetacoplan reduced the rate of GA lesion growth compared with sham treatment. Geographic Atrophy is considered a late-stage form of dry AMD [22,23,24]. RP, caused by mutations in over 90 genes, affects approximately 1 in 4000 individuals and typically progresses from night blindness to progressive visual-field loss and, in many patients, severe vision loss, with substantial variation according to genotype and individual disease course (https://retnet.org/) [3,25]. Stargardt disease (prevalence ~1 in 8000–10,000) results from biallelic ABCA4 mutations driving toxic bisretinoid accumulation in the RPE, causing juvenile-onset macular atrophy [26]. DME affects approximately one-third of diabetic patients with >20 years of disease duration, and RVO is the second most common retinal vascular disorder after diabetic retinopathy [20,27,28], both causing macular cell loss that anti-VEGF therapy can slow but not reverse. The combined economic, social, and psychological toll of these conditions, including loss of independence, reduced employment, and diminished quality of life, underscores the urgent need for therapies that go beyond symptom management to true cellular restoration.

### 1.2. Current Therapeutic Strategies for Retinal Diseases

#### 1.2.1. Cell Therapy Overview: RPE and Photoreceptor Transplantation

Cell-based therapy seeks to replenish lost or dysfunctional retinal cells through transplantation of exogenous counterparts, with RPE cells and photoreceptors representing the two principal therapeutic targets. Early clinical trials using hESC-derived RPE demonstrated acceptable safety profiles and exploratory anatomical and functional outcomes, including variable improvements in best-corrected visual acuity and quality-of-life measures; however, these phase I/II studies were not designed to establish efficacy in AMD and Stargardt patients [5,6]. In addition, a landmark phase I/IIa trial demonstrated that a bioengineered RPE monolayer on a synthetic scaffold could be surgically implanted and was well tolerated in patients with dry AMD, without serious adverse events, supporting the feasibility of RPE replacement strategies [11]. More recently, Masayo Takahashi’s group at RIKEN completed the first-in-human autologous iPSC-derived RPE transplantation in a neovascular AMD patient, establishing the feasibility of the fully autologous approach [29]. Several trials are currently in phase I/II, including those by the London Project to Cure Blindness and Astellas (formerly Ocata Therapeutics) [5,30].

#### 1.2.2. iPSC Technology: Advantages and Patient-Specific Therapy

The discovery by Takahashi and Yamanaka that adult somatic cells can be reprogrammed into pluripotent stem cells using defined transcription factors transformed the landscape of regenerative medicine [7]. For retinal disease specifically, iPSCs can be directed toward RPE, photoreceptor precursors, and retinal organoids through stage-specific differentiation protocols that recapitulate embryonic retinogenesis [31,32]. Patient-specific iPSC-derived RPE has been used to model Stargardt disease in vitro, revealing ABCA4-dependent lipofuscin accumulation as a druggable target [33], and iPSC-derived photoreceptors have been transplanted in animal models of RP with evidence of outer nuclear layer integration [32].

#### 1.2.3. CRISPR Gene Editing: Mechanism and Retinal Applications

CRISPR-Cas9 enables targeted double-strand breaks at virtually any genomic locus using a programmable single guide RNA, allowing correction, disruption, or insertion of genetic sequences with unprecedented precision [9]. In retinal disease, key pathogenic mutations targeted include *ABCA4* (Stargardt disease), *RPGR* and *PRPH2* (X-linked and autosomal RP), and *CEP290* (Leber congenital amaurosis), collectively accounting for a substantial proportion of inherited retinal dystrophy cases [2,34]. CRISPR has been used to correct *ABCA4* mutations in patient-derived iPSCs modeling Stargardt disease, repair pathogenic *RPGR* frameshift mutations in X-linked RP patient-derived retinal organoids, disrupt a deep intronic *CEP290* mutation, and edit *PRPH2* haploinsufficiency in photoreceptor precursors [35,36,37]. The *CEP290* approach has advanced furthest clinically, with EDIT-101 (Editas Medicine, NCT03872479) representing the first in vivo CRISPR trial in the human eye [38].

#### 1.2.4. Combination Approach: Gene Correction, Differentiation, and Transplantation—An Integrated Pipeline

The most clinically ambitious strategy integrates all three technologies sequentially: patient-derived cells are reprogrammed into iPSCs, CRISPR-corrected, differentiated into the target retinal cell type, and delivered to the subretinal or suprachoroidal space via specialized surgical systems [10,32]. Individual components of this pipeline have been demonstrated preclinically for RPE and photoreceptor targets, and partial implementations have entered early clinical testing (for example, autologous iPSC-derived RPE without gene correction), whereas a fully integrated, gene-corrected autologous pipeline has not yet been clinically demonstrated; such an integrated pipeline would represent the most complete embodiment of precision regenerative ophthalmology [11,29].

### 1.3. Limitations of Current Therapies

Despite the progress outlined above, all three therapeutic modalities face fundamental and partially overlapping limitations that have thus far prevented curative outcomes at clinical scale. Gene therapy using Adeno-associated virus (AAV) vectors has achieved landmark regulatory successes, most notably Voretigene neparvovec (Luxturna) for RPE65-associated Leber congenital amaurosis [39]. However, this approach remains inherently restricted to single-gene disorders with known causative mutations and accessible target cells. AAV capsid immunogenicity can complicate or limit repeat administration, the ~4.7 kb packaging capacity limits delivery of very large genes such as *ABCA4* and *RPGR* (for which truncated or codon-optimized constructs have nevertheless been investigated clinically) [40], and the theoretical risk of insertional mutagenesis raises long-term safety concerns. Critically, gene therapy can preserve or rescue surviving cells but cannot restore those already lost, rendering it ineffective in patients presenting with advanced degeneration [40]. Similarly, cell-based therapies depend on a clinical therapeutic window, as sufficient inner retinal integrity, macular architecture, and/or residual photoreceptors must be present for transplanted cells or grafts to achieve functional benefit.

Cell therapy directly addresses irreversible cell loss but introduces a distinct set of translational barriers. Whether transplanted cells achieve true synaptic integration with host retinal circuitry, or whether observed visual gains reflect paracrine neuroprotection rather than genuine circuit reconstruction, remains unresolved, a distinction with profound implications for the ceiling of functional recovery [41]. Manufacturing iPSC-derived retinal cells to the scale, purity, and consistency required for clinical use is technically demanding and expensive; batch-to-batch variability in differentiation efficiency and cell identity poses both safety and efficacy concerns [10,42]. Immunological rejection of allogeneic grafts requires systemic immunosuppression carrying its own risks [12]. Autologous iPSC-derived grafts may reduce, but do not eliminate, immune-mediated rejection risk, and they shift the burden to patient-specific manufacturing, cost, and verification of the genomic integrity of each line [8,29]. While surgical delivery of fragile cell suspensions or sheets into the delicate subretinal space demands extraordinary precision, off-target deposition can cause retinal detachment or inflammatory reactions [10,11]. The iPSC-specific limitations add further complexity: inter-line variability in differentiation efficiency remains poorly predictable, residual epigenetic memory from the somatic donor cell can bias lineage commitment, and no reliable non-destructive assay currently exists to certify a given iPSC line as clinically fit-for-purpose before committing it to a full manufacturing run [43]. For CRISPR-based approaches, despite the precision of guide RNA targeting, off-target cleavage at genomically similar loci, large chromosomal deletions, and p53-mediated genotoxic stress responses have all been documented in edited pluripotent cells. No consensus screening standard has yet been established for clinical-grade iPSC clone release following gene editing [17,44].

Collectively, these limitations, spanning biological, manufacturing, surgical, and regulatory dimensions, underscore that no single technology is sufficient in isolation, and that their integration into a reliable, scalable pipeline remains the central unsolved challenge of regenerative retinal medicine, where AI-based approaches may play an enabling role in improving quality control, predictive differentiation modeling, and safety screening across iPSC and gene-edited cell manufacturing workflows.

## 2. Artificial Intelligence as a Support Tool in Retinal Medicine

AI, broadly encompassing ML, DL, and computer vision, and implemented through CNNs, transformer architectures, and generative models, has rapidly become one of the most consequential methodological advances in modern medicine. In ophthalmology specifically, AI has found particularly fertile ground: retinal imaging generates large, structured, high-resolution datasets with well-defined visual phenotypes, creating ideal conditions for supervised learning [13,14]. DL systems trained on fundus photographs, optical coherence tomography (OCT), OCT angiography (OCTA), and fluorescein angiograms (FA) have demonstrated diagnostic accuracy rivaling or exceeding expert clinicians for AMD, diabetic retinopathy, glaucoma, and retinal vein occlusion in retrospective evaluations, with some models diagnosing over 50 retinal conditions simultaneously [13,14,15].

Beyond diagnosis, AI is being applied to disease progression monitoring, pharmacological response prediction, surgical planning, and multimodal biomarker discovery, capabilities that extend its utility well beyond a diagnostic adjunct [45,46]. In the context of cell-based therapies specifically, AI has been proposed or explored as a support tool across the entire therapeutic pipeline: stratifying patients most likely to benefit from transplantation, optimizing iPSC culture and differentiation, ensuring manufacturing quality control, guiding intraoperative surgical delivery, and monitoring post-transplantation graft survival and functional integration [16,17,42,45]. Crucially, AI integration into these workflows does not supplant clinical or biological judgment; it augments it, providing quantitative, reproducible, and scalable analytical capabilities that may be difficult to achieve by conventional means.

### Challenges of AI in Retinal Medicine and Cell Therapy

Despite its promise, AI faces distinct challenges in retinal diagnostics versus cell-based therapy workflows. In retinal imaging, the principal constraint is data scarcity and standardization: labeled datasets for rare conditions such as Stargardt disease or specific RP subtypes are geographically concentrated and rarely harmonized across imaging platforms, while distributional shifts from differences in OCT hardware and patient demographics degrade model performance outside training environments [14,15,47]. In cell therapy manufacturing, the challenge is more severe. Training datasets for iPSC morphology classifiers, bioreactor process models, and CRISPR off-target predictors are predominantly proprietary, small in scale, and generated under variable experimental conditions, making independent validation exceptionally difficult [48]. Across both domains, interpretability remains a shared barrier: most state-of-the-art DL models function as black boxes, and while explainability techniques such as Grad-CAM, SHAP, and attention visualization have partially addressed this, they remain insufficient for high-stakes decisions such as releasing a cell therapy batch or approving a CRISPR-edited clone for clinical use [14,15,49].

Regulatory and ethical challenges compound these technical barriers. While the FDA has issued guidance on Software as a Medical Device (SaMD) [50,51] and the European Medicines Agency (EMA) has issued a reflection paper on AI in the medicinal product lifecycle [50], a mature, dedicated, harmonized regulatory framework specifically addressing continuously learning or AI-dependent components embedded within cell therapy manufacturing processes or intraoperative surgical systems has not yet been established, although existing medical-device, software, medicinal-product, combination-product, GMP, and quality-system frameworks may nevertheless apply. These are contexts where AI outputs directly determine patient safety rather than merely informing clinical judgment [45]. Prospective clinical evidence demonstrating safety and efficacy of AI-guided cell therapy workflows remains largely absent. AI models trained predominantly on data from high-income, European-ancestry populations risk poor performance in underrepresented groups, potentially exacerbating existing disparities in access to advanced retinal care [13,14], a challenge that is as much ethical as it is scientific.

## 3. Literature Search and Scope

### 3.1. Search Strategy

A comprehensive literature search was conducted across PubMed, Scopus, and Web of Science, with the primary search covering publications from January 2015 to March 2025, a period reflecting the emergence of substantive AI applications in cell-based therapeutic workflows. A subsequent update search was performed to identify relevant recent literature; the final search date was 23 September 2026. The following Boolean search strategy was applied across all three databases:

(*“artificial intelligence” OR “machine learning” OR “deep learning” OR “neural network” OR “computer vision” OR “reinforcement learning”*) AND (*“retinal disease” OR “age-related macular degeneration” OR “retinitis pigmentosa” OR “Stargardt disease” OR “diabetic retinopathy” OR “diabetic macular edema” OR “retinal vein occlusion”*) AND (*“cell therapy” OR “cell-based therapy” OR “stem cell” OR “iPSC” OR “induced pluripotent stem cell” OR “retinal pigment epithelium” OR “photoreceptor” OR “CRISPR” OR “gene editing” OR “retinal organoid”*).

The update search used the Boolean strategy above, unrestricted by field tags, across all three databases. It retrieved 206 records from PubMed, 565 from Scopus, and 218 from Web of Science (989 records total), which reduced to 646 unique records after de-duplication by DOI and, where no DOI was available, by normalized title. Of these, 441 records were excluded at title/abstract screening (chiefly AI-based retinal imaging/diagnostics without a cell-therapy component, *n* = 279; pharmacological or anti-VEGF therapy without a cellular component, *n* = 62; ineligible publication type such as conference papers, editorials, book chapters, notes, or letters, *n* = 78; non-English language, *n* = 11; and cell-therapy topic without an AI/ML technique, *n* = 11). The remaining 205 records underwent closer assessment against the eligibility criteria in Section 3.2, of which 129 were excluded: 76 were found, on reading the available abstract, to have no discernible AI/ML component (these had been retrieved via the retinal-disease and cell/gene-editing search terms rather than the AI search terms) and 53 could not be confirmed as eligible because no abstract was available and the title gave no indication of an AI/ML method, so these were conservatively excluded. This yielded 76 included records: 28 addressing AI applications directly within a cell-based therapeutic workflow stage, and 48 addressing AI applications in retinal imaging or diagnostics that are retinal-adjacent rather than directly cell-therapy-focused; the latter are cited in this review as contextual, retinal-adjacent evidence rather than direct cell-therapy evidence, consistent with the evidence-level framework described in Section 4. The recent studies identified by this update search are integrated in the relevant subsections of Section 4.

Foundational technology papers predating 2015, including seminal studies on iPSC reprogramming [7], CRISPR-Cas9 mechanism [9], and early-phase hESC-RPE clinical trials [5,6], were identified through systematic citation chaining (backward and forward reference tracking of key articles and seminal studies) and targeted hand-searching of papers. These studies were included as essential background references irrespective of their publication date. Recent studies, preprints, and proof-of-concept studies identified by citation chaining or hand-searching outside the primary search window were included when they provided the primary evidence for a specific claim or directly informed the pipeline stage under discussion; preprints and conference abstracts are explicitly identified as such in the reference list and were not used as the sole basis for strong clinical or biological conclusions. Clinical trial data were additionally sourced from ClinicalTrials.gov, restricted to trials registered between January 2015 and December 2025, as summarized in Table 1 (search date and search terms are given in the note to Table 1).

### 3.2. Inclusion and Exclusion Criteria

Articles were considered eligible if they addressed the application of at least one AI technique, encompassing ML, DL, computer vision, reinforcement learning, or natural language processing, to any stage of a cell-based therapeutic workflow for retinal disease. Eligible workflow stages included patient stratification, iPSC culture and quality control, gene editing safety assessment, retinal organoid characterization, cell manufacturing and bioprocess optimization, intraoperative surgical guidance, and post-transplantation outcome monitoring. Given the interdisciplinary scope of this review, studies addressing AI applications in adjacent domains, including retinal image analysis, biological process optimization, and surgical robotics, were also included where directly relevant to the cell therapy pipeline.

Articles were excluded if they: addressed pharmacological, laser, or anti-VEGF therapies without a cellular transplantation component; involved non-retinal ocular tissues exclusively; were limited to AI diagnostics without any connection to a cell-based therapeutic workflow; or were conference abstracts, editorials, or non-peer-reviewed reports (with limited exceptions for recent preprints and conference or supplement abstracts that provided the only available evidence for an emerging application, which are labeled as such in the reference list). Non-English language publications were also excluded. Original research articles, preclinical studies, proof-of-concept investigations, and early-phase clinical studies were all considered eligible, reflecting the early translational stage of AI integration in this field.

### 3.3. Source Selection

This review is an integrative narrative review and does not follow PRISMA reporting guidelines. An integrative narrative approach was chosen because of the breadth of technologies covered, spanning stem cell biology, gene editing, biological manufacturing, surgical robotics, and retinal imaging, and the heterogeneity of study designs and endpoints across these domains, which favored a conceptual synthesis over a formal systematic or scoping methodology, although such approaches could be applied to individual pipeline stages in future work. Instead, source selection prioritized studies that were methodologically rigorous, clinically relevant, and representative of the current state of evidence within each pipeline domain. Where evidence was sparse, particularly for intraoperative AI guidance and bioreactor optimization, preclinical and proof-of-concept studies were included to map the trajectory of the field rather than its current ceiling. Seminal papers in foundational technologies, including iPSC reprogramming [7], CRISPR-Cas9 gene editing [9], and hESC-RPE transplantation [5,6], were included regardless of publication date given their direct and indispensable relevance to the review scope.

## 4. AI Enhancing Cell Therapy and Its Key Applications

The integration of AI across the cell-based retinal therapy pipeline spans a wide spectrum of technical approaches and translational stages. Table 2 provides a structured overview of key AI applications across the 10 pipeline domains reviewed, including the AI architecture employed, the retinal disease or cell type addressed, and the primary outcome reported, together with the evidence level, study setting, sample size, and external-validation status of each entry (Table 2A,C).

Each entry in Table 2A,C is classified by evidence level as follows: (i) direct retinal cell-therapy evidence (studies using retinal cell products or transplanted retinal cells); (ii) retinal-adjacent evidence (AI applied to retinal imaging, retinal disease, retinal organoids, or retinal gene therapy without a cell-therapy product); (iii) cross-domain proof of concept (AI applied to generic iPSC or cell manufacturing, non-retinal transplantation, or other non-retinal biological systems, which may be transferable but has not been demonstrated in retinal cell therapy); and (iv) conceptual or proposed only (no supporting study identified in the retinal cell-therapy setting). Performance metrics are reported as in the primary studies; because tasks, populations or model systems, validation settings, and metrics differ, they are not directly comparable across rows. Preprints are labeled as such.

The following subsections synthesize evidence across 10 key domains in which AI has been applied, or proposed for application, to cell-based retinal therapies, progressing from patient-facing diagnostic applications through to manufacturing, surgical delivery, and post-transplantation monitoring.

### 4.1. AI in Retinal Imaging and Disease Monitoring

Retinal imaging is the cornerstone of ophthalmological diagnosis and monitoring, encompassing a growing armamentarium of modalities including color fundus photography, spectral-domain OCT, OCT angiography (OCTA), fundus autofluorescence (FAF), and adaptive optics scanning laser ophthalmoscopy (AOSLO). Each modality captures distinct aspects of retinal structure and function, and their integration provides a comprehensive view of disease state. AI, particularly deep CNNs and vision transformer architectures, has shown strong performance in retrospective studies across several of these modalities [13,14,15]. DL models have achieved near-expert-level accuracy in detecting and quantifying drusen, geographic atrophy, and neovascular lesions from OCT and fundus images, with some architectures diagnosing over 50 retinal conditions simultaneously [13,14,15]. Longitudinal AI-powered analysis can support tracking of lesion progression and measurement of geographic atrophy growth rates [74]. In RP, AI-based OCT and FAF analysis has enabled automated mapping of the preserved ellipsoid zone, outer nuclear layer, and autofluorescence abnormalities, providing sensitive biomarkers of disease progression that could serve as quantitative endpoints in future cell therapy clinical trials [71,72].

**Figure 1:**
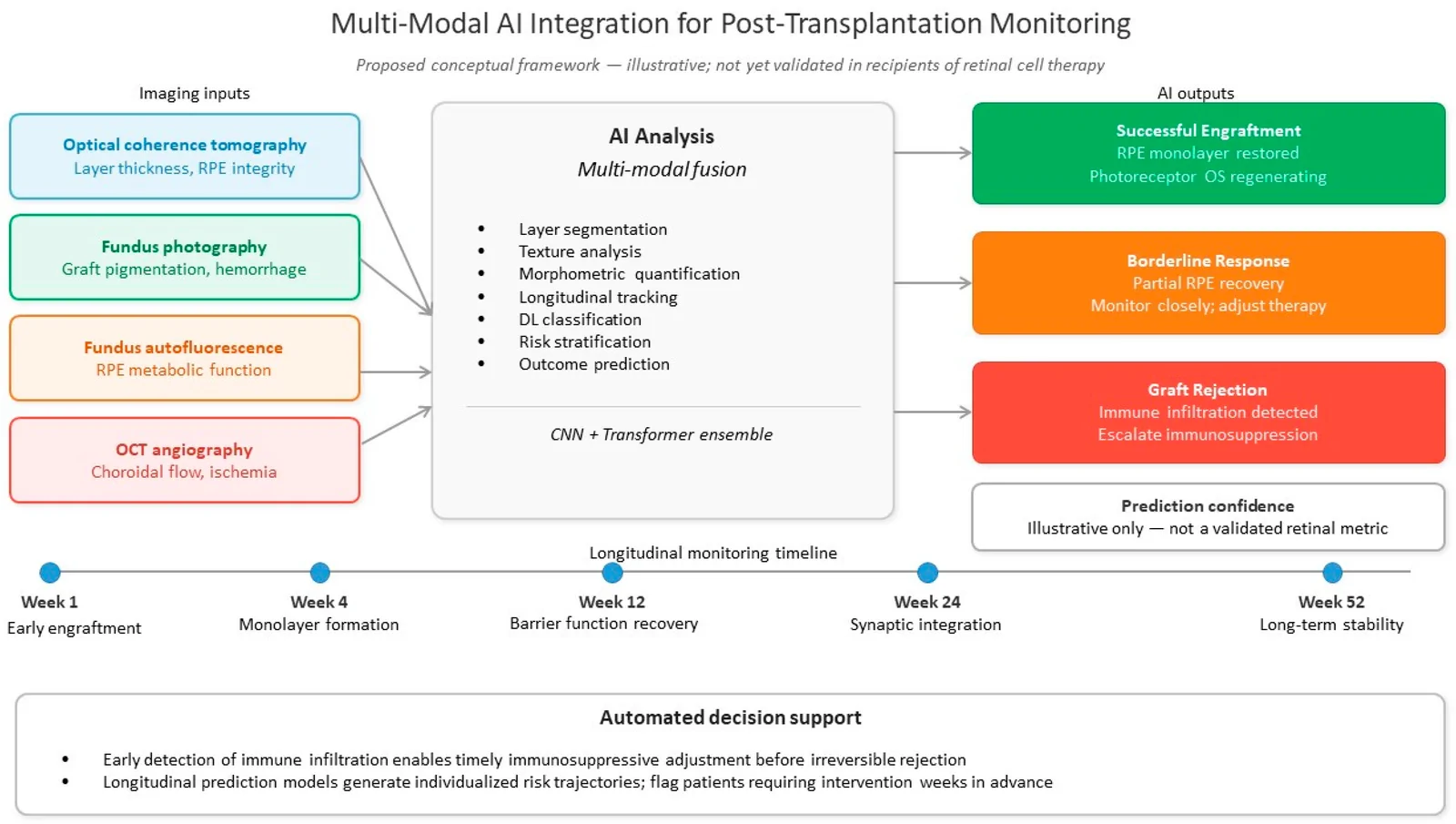
Proposed conceptual multi-modal AI integration framework for post-transplantation retinal monitoring. In this framework, AI systems fuse information from OCT, fundus photography, fundus autofluorescence, and OCT angiography to detect RPE recovery, photoreceptor regeneration, and immune responses, generating risk-stratified predictions across the first 52 weeks post-transplant. The framework is illustrative and has not been validated in recipients of retinal cell therapy; no performance value for retinal graft classification is implied. For reference, an AUC of up to 0.92 has been reported only for kidney-graft failure prediction in an analogous solid-organ setting [69]. Early detection could, in principle, enable timely clinical intervention.

In the context of post-transplantation monitoring (Figure 1), AI-powered multimodal image analysis is proposed to be well suited to detect subtle graft-related changes, including engraftment of transplanted RPE sheets, photoreceptor outer segment regeneration, immune infiltration, or sub-RPE fluid accumulation, that may be imperceptible by conventional clinical grading [11,45]. This application is extrapolated from retinal-adjacent imaging studies and has not been validated in recipients of retinal cell transplants. Longitudinal multi-visit models integrating baseline and post-operative imaging with patient demographic and genetic data could provide a holistic view of transplantation outcomes and may generate individualized prognosis estimates to inform clinical management decisions.

### 4.2. AI in Stem Cell Biology and iPSC Quality Control

The reproducibility and safety of iPSC-derived cell products depend critically on the quality of input iPSC lines before any differentiation is attempted. Conventional quality control methods, including karyotyping, immunofluorescence for pluripotency markers such as OCT4, SOX2, and NANOG, and embryoid body formation assays, are labor-intensive, time-consuming, and provide only categorical rather than continuous quality assessments that are poorly suited to large-scale clinical manufacturing [75]. CNNs and other ML classifiers trained on iPSC colony brightfield or phase-contrast images have been used to classify colony quality and pluripotency status, and to predict the early onset of differentiation, from morphology, with reported accuracies of approximately 85–99% depending on the study, model, and ground-truth definition [54,55,57,62]. In these studies, the models were trained against morphological or marker-based labels (for example, expert-annotated colony quality or pluripotency status); direct prediction of genomic stability, tumorigenicity, or clinical release suitability was not the trained endpoint. Morphology should therefore be regarded as a surrogate that must be distinguished from molecular identity, genomic integrity, tumorigenicity risk, and formal release testing. Such models can flag colonies exhibiting morphological aberrations, including irregular borders, vacuolation, and differential growth patterns, that may reflect partial differentiation or other culture-quality problems; a relationship with chromosomal instability has not been established by the cited studies and would require karyotypic or sequencing-based ground truth. If validated in this way, such models could support continuous non-invasive culture monitoring without sacrificing material for molecular assays.

Beyond colony-level morphological assessment, AI is being applied to characterize the transcriptional landscape of iPSC populations at single-cell resolution. Graph neural networks and variational autoencoders have been used for clustering and rare-population inference in scRNA-seq data [58,60]. A bulk-transcriptomic deconvolution algorithm assigned only 0–5% of signal to an “undetermined” category in leave-one-out benchmarking, even when a cell type was fully omitted from the reference; this describes deconvolution accuracy, not the prevalence of rare iPSC subpopulations [59]. Neither approach has directly demonstrated detection of clinically dangerous iPSC subpopulations, such as tumorigenic or aberrant clones. Separately, rare subpopulations in iPSC cultures have been reported to display transcriptional profiles linked to malignant transformation, aberrant lineage commitment, and impaired differentiation competence, underscoring their potential relevance to iPSC safety, quality control, and clinical translation [76]. Single-cell approaches can resolve populations that bulk transcriptomic analyses cannot, which is relevant to stem cell manufacturing. If such methods were validated against orthogonal safety assays (for example, genomic integrity and tumorigenicity testing), iPSC clones containing high-risk subpopulations could potentially be identified and excluded prior to large-scale expansion or differentiation, which might reduce safety concerns and improve manufacturing efficiency; this has not yet been demonstrated. Such early-stage quality control measures are particularly important for clinical-grade cell production, where the presence of rare aberrant cells may compromise therapeutic efficacy or increase the risk of adverse outcomes. Nevertheless, it is important to note that while iPSCs represent the predominant cell source in current retinal cell therapy development, other stem cell populations, including hESC-derived RPE [5,6], retinal progenitor cells, and Müller glia-derived progenitors, are also under active investigation. However, the availability of large, well-annotated validation datasets for non-iPSC populations remains limited, highlighting the need for further studies to establish the robustness, generalizability, and regulatory applicability of AI-based quality assessment across diverse retinal cell therapy products.

### 4.3. AI in Optimization of Retinal Cell Differentiation

Once iPSC lines have been quality-certified, directed differentiation into retinal cell types represents the next critical and technically demanding stage of the manufacturing pipeline. Differentiation protocols for RPE cells, photoreceptor precursors, and retinal ganglion cells involve complex multi-stage sequences spanning four to twelve weeks, during which cells are exposed to precisely timed growth factors, small molecules, and extracellular matrix cues that recapitulate embryonic retinogenesis [31,32]. Minor deviations in growth factor concentrations, temperature fluctuations, or cell seeding density can substantially alter differentiation efficiency, cell identity, and final product purity, introducing variability that conventional fixed protocols cannot adequately control. Reinforcement learning (RL) has been proposed as a strategy for optimizing multi-step differentiation protocols, using different doses of differentiation factors, by treating them as sequential decision problems in which an agent receives feedback based on the state of the culture at each timepoint. A recent preprint describes such a Differentiation with Reinforcement Learning (DiRL) framework, in which RL agents select sequential genetic perturbations within a single-cell foundation model; it was evaluated computationally on iPSC-derived organoid datasets and outperformed random perturbation baselines with a 69% win rate [61]. This is a non-peer-reviewed, computational proof of concept. It has not been shown experimentally to increase RPE or photoreceptor purity, and it should not be interpreted as evidence that RL-controlled retinal differentiation protocols improve product purity.

Image-based ML analysis of phase-contrast and fluorescence microscopy images captured at defined differentiation timepoints has been reported to predict differentiation outcome before the run is complete. In retinal systems, CNNs applied to bright-field images have predicted retinal differentiation in mouse embryonic stem cell-derived retinal organoids before reporter expression became detectable [77], and deep learning has predicted the emergence of RPE tissue in medaka retinal organoids [64]. Evidence in non-retinal lineages, such as iPSC-derived muscle stem cells [78], supports the transferability of this concept but is not retinal-specific. Such predictive models could allow timely termination of low-quality batches, reducing wasted resources and manufacturing timeline, and could be integrated into closed-loop automated culture platforms that dynamically adjust protocol parameters in response to real-time quality signals [78]. In principle, AI-based differentiation monitoring could apply across target cell types, because RPE, photoreceptor precursor, and retinal ganglion cell protocols each generate distinct morphological and transcriptional trajectories that AI models could be trained to recognize. For RPE, deep learning has been used to predict the function of live iPSC-derived RPE from quantitative microscopy [79], whereas equivalent evidence for photoreceptor precursor and retinal ganglion cell products remains limited.

### 4.4. AI in CRISPR Gene Editing Safety

The safety and efficacy of CRISPR-Cas9 editing in iPSCs depend critically on guide RNA (gRNA) design that maximizes on-target cleavage while minimizing off-target activity. Conventional bioinformatic tools rely on sequence-based scoring algorithms with limited ability to capture complex interactions between gRNA sequence, chromatin accessibility, DNA repair pathway engagement, and Cas9 binding kinetics. ML models trained on large empirical datasets of gRNA-target interactions have substantially improved predictive accuracy of both on-target and off-target scores [80]. DL architectures including bidirectional recurrent neural networks, convolutional sequence models, and transformer-based models trained on genome-wide CRISPR screen data demonstrate superior performance in predicting editing efficiency across diverse genomic contexts. Some of these models incorporate epigenetic features, such as chromatin accessibility and histone-mark data, to enable more context-specific predictions [81]; whether they capture the epigenetic landscape of iPSCs, which may differ substantially from the cell types on which many training datasets are based, has not been established. For off-target detection, variant-aware search tools with ML-based activity scoring, such as VARSCOT, take individual genomic variation into account and were reported to identify 40–70% more experimentally verified off-target sites than other popular software tools [53]; this was an in silico benchmark and not an analysis of whole-genome sequencing data from edited iPSC clones. Whole-genome sequencing-based screening of edited clones for unintended edits, chromosomal rearrangements, and large deletions is an established safety concern [82]; the use of AI for this purpose in iPSC clone release is a proposed application rather than a demonstrated one. This reflects improved detection sensitivity rather than a reduction in the biological occurrence of off-target editing, and improved detection does not eliminate the need for empirical safety testing. AI-based gRNA design and off-target prediction tools are widely used in research settings, but their routine adoption in clinical-grade iPSC editing workflows has not been systematically documented.

### 4.5. AI in Retinal Organoid Development and Drug Screening

Retinal organoids are three-dimensional self-organizing iPSC-derived structures that recapitulate key aspects of in vivo retinal development. They have emerged as powerful tools for disease modeling, drug screening, and the study of retinal cell-cell interactions [31,83]. However, individual organoids within the same culture vary substantially in size, lamination, cell type composition, and maturation state, introducing significant challenges for systematic characterization. DL-based image analysis applied to brightfield, fluorescence, and light-sheet microscopy images has been used for automated segmentation of organoid boundaries, quantification of laminar organization, and identification of specific cell types based on morphological and fluorescence signatures, without the need for destructive molecular assays [63,64]. These studies were performed in gastruloids and other multilayered organoids [63] and in medaka retinal organoids [64]; their applicability to human iPSC-derived retinal organoids is therefore inferred rather than demonstrated. Generative models including variational autoencoders and GANs have been applied to learn latent representations of cell morphological states (demonstrated in non-retinal cell-morphology profiling of drug responses [65]), which could enable continuous phenotypic scoring and unsupervised clustering of organoids by biological similarity.

In drug screening applications, AI-powered organoid analysis may help identify compounds modulating retinal cell survival, differentiation, or function in disease-relevant models, potentially accelerating the identification of candidate therapeutics for clinical translation; evidence for screening in retinal organoids specifically remains limited (Table 2). Furthermore, AI analysis of single-cell RNA-sequencing data from patient-derived retinal organoids further enables identification of cell-type-specific transcriptional disease signatures, providing mechanistic insights that guide the design of targeted therapeutic strategies [84,85].

### 4.6. AI in Cell Manufacturing and Bioreactor Optimization

The clinical translation of iPSC-derived cell therapies requires scalable, reproducible, and cost-effective manufacturing processes. Current platforms, including planar culture systems and stirred-tank bioreactors, are characterized by significant batch-to-batch variability driven by differences in environmental conditions, raw material quality, and operator technique. AI-powered process monitoring and control may represent a step toward more autonomous, adaptive bioprocessing [86,87].

Evidence specific to retinal cell products is more limited than the general bioprocessing literature. Stirred-tank bioreactor culture has been shown to improve laminar stratification and photoreceptor yield in human retinal organoids, although without AI-based process control [88], and image-based deep learning has been used to predict the function of live iPSC-derived RPE, a retinal cell product, in vitro [79]. By contrast, the LSTM-based process-modelling study discussed below concerns a HEK293 production process [67], and the iPSC bioreactor studies [87,89] concern generic iPSC platforms and are published as conference abstracts. We therefore distinguish (i) retinal-specific manufacturing studies [79,88], (ii) generic iPSC manufacturing studies [87,89], and (iii) non-retinal or unrelated production systems [67,90]; methods in categories (ii) and (iii) are presented as transferable concepts rather than as evidence of established retinal-cell manufacturing performance.

In bioreactor systems, arrays of inline sensors continuously monitor dissolved oxygen tension, pH, osmolality, glucose, and lactate concentrations, thus generating dense time-series data reflecting the metabolic state of the culture. Machine learning models, particularly LSTM, recurrent networks and physics-informed neural networks trained on historical bioreactor run data have been used to predict future culture trajectories, as demonstrated for a HEK293 production process [67]. In principle, the same approach could identify early signatures of suboptimal cell growth or metabolic stress ahead of process failure in retinal cell manufacturing, but this has not been demonstrated for RPE or photoreceptor products. Computer vision systems integrated with bioreactor viewing ports can provide real-time morphological monitoring, complementing biochemical sensor data with spatial information. Digital twin frameworks, computational models simulating a specific bioreactor run in real time, could enable virtual scenario testing of feeding strategies, gas control, and agitation parameters without perturbing physical cultures, potentially reducing process development iteration cycles and accelerating protocol lock for clinical manufacturing [90,91]. Thus, these AI-enhanced manufacturing systems may hold the potential to reduce the cost and improve the reliability of clinical-grade cell production, which could help bring autologous and allogeneic cell therapies closer to broad clinical deployment; this remains to be demonstrated for retinal cell products.

### 4.7. AI in Surgical Delivery and Post-Transplantation Monitoring

The surgical delivery of cell therapies to the retina demands extraordinary precision: subretinal injection of fragile cell suspensions or sheets must achieve accurate anatomical placement without causing retinal perforation, detachment, or inflammatory reactions. Computer vision algorithms integrated with intraoperative OCT and surgical microscopes are being developed to provide real-time anatomical guidance, delineating the subretinal space, identifying residual photoreceptor layers, and flagging areas of retinal elevation or trauma during injection [45]. Robot-assisted delivery systems can reduce tremor and improve injection precision. In a randomized preclinical study in 48 pigmented rabbits (24 per group), robot-assisted subretinal injection achieved a first-attempt success rate of 91.7% (22/24) versus 37.5% (9/24) with manual injection [68]. This result derives from an animal model, did not involve human cell transplantation, and has not yet been replicated in human subretinal cell transplantation. AI analysis of post-injection OCT images immediately following cell delivery could assess the distribution and volume of the transplanted bolus, providing intraoperative feedback to guide additional injection if target coverage is inadequate.

Post-operatively, DL models trained on longitudinal imaging datasets could in principle distinguish features associated with successful engraftment, including restoration of the RPE monolayer autofluorescence signal, emergence of photoreceptor outer segment reflectivity, and normalization of choroidal vasculature, from features of graft failure or rejection, including subretinal fluid accumulation and inflammatory infiltration, an application that is proposed here and has not been demonstrated in recipients of retinal cell therapy; it builds on retinal-adjacent evidence for expert-level diagnostic performance from OCT-based retinal biomarkers, including DL-based OCT layer segmentation in patients undergoing RPE65 gene therapy [14,70]. As an external analogy, a machine-learning model trained on retrospective clinical data from deceased-donor kidney transplant recipients reached an AUC of up to 0.92 for graft-failure prediction in the 9–15-month post-transplant window, with confirmation in a blinded external validation cohort [69]. This value derives from a different organ, data modality, and clinical setting; it should not be interpreted as an expected performance for retinal graft monitoring, and comparable performance has not been demonstrated for RPE or photoreceptor cell therapy. These models could in principle generate probabilistic predictions of long-term outcomes from early post-operative imaging findings, enabling individualized risk stratification and timely clinical intervention when adverse trajectories are detected.

## 5. The AI-Powered Cell Therapy Pipeline

**Figure 2:**
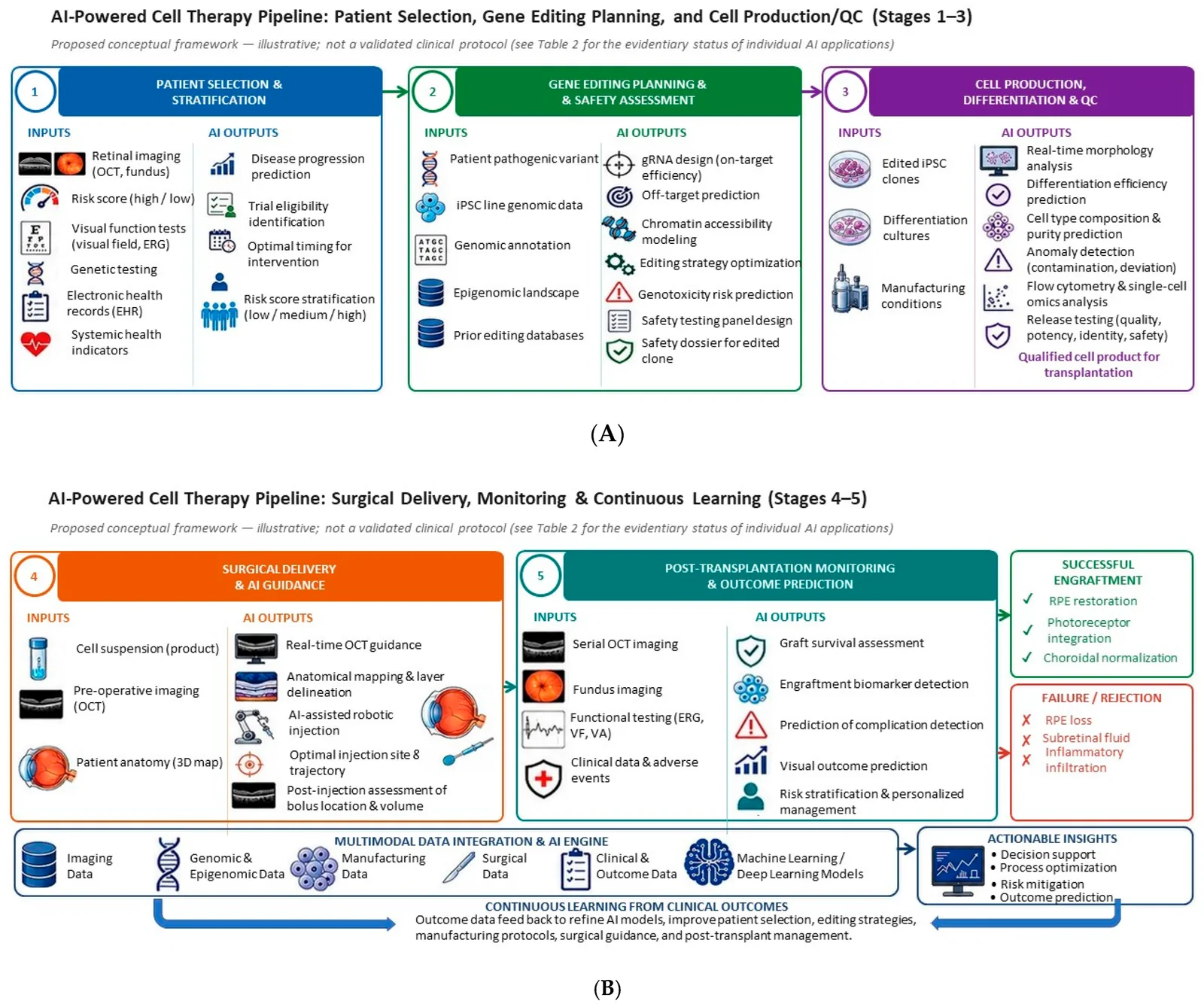
(**A**) AI-powered cell therapy pipeline: patient selection and stratification, gene editing planning and safety assessment, and cell production, differentiation, and quality control (pipeline Stages 1–3). This is a proposed conceptual framework; the stages, inputs, and AI outputs shown are illustrative and forward-looking and do not represent a clinically validated pathway. Where supporting evidence exists, it derives predominantly from retinal-adjacent, cross-domain, or preclinical studies (Table 2A; Section 4.1, Section 4.2, Section 4.3, Section 4.4, Section 4.5, Section 4.6 and Section 4.7). (**B**) AI-powered cell therapy pipeline: surgical delivery and AI guidance, post-transplantation monitoring and outcome prediction, and the continuous-learning integration loop (pipeline Stages 4–5). This is a proposed conceptual framework; the stages, inputs, and AI outputs shown are illustrative and forward-looking and do not represent a clinically validated pathway. Where supporting evidence exists, it derives predominantly from retinal-adjacent, cross-domain, or preclinical studies (Table 2A; Section 4.1, Section 4.2, Section 4.3, Section 4.4, Section 4.5, Section 4.6 and Section 4.7).

The preceding sections have described AI applications within individual stages of the cell therapy workflow. Realizing the potential of AI may require its integration across the entire continuum from patient selection to post-transplantation monitoring, creating an interconnected pipeline in which data and insights generated at each stage inform and optimize subsequent steps. Crucially, Section 5 emphasizes the integration between stages: how superior patient selection (Section 5.1) could enable less intensive monitoring (Section 5.5), how successful manufacturing quality (Section 5.3) could predict surgical outcomes (Section 5.4), and how post-operative performance (Section 5.5) could retroactively validate manufacturing and patient selection decisions (feeding back to Section 5.2 and Section 5.3). In this respect, a proposed framework/model could be as presented in Figure 2. The framework presented in this section is a hypothetical, proposed conceptual framework developed by the authors; it is not a validated clinical pathway or protocol. The stages, assessment time points, risk tiers, thresholds, and decision rules described below are illustrative, forward-looking elements intended to show how information could flow between stages; unless a specific reference is cited, they are not derived from prospectively validated clinical-trial data or guidelines for retinal cell therapy and should not be used to guide clinical management (Figure 2A,B).

### 5.1. Stage 1: Patient Selection and Stratification

The first and arguably most consequential step in the cell therapy pipeline is the identification of patients who are most likely to derive meaningful benefit from treatment, and who are at sufficiently advanced but not irreversible stages of disease to warrant intervention. AI-based patient stratification could integrate multimodal clinical data including retinal imaging, visual function testing (visual acuity, visual field, electroretinography), genetic mutation status, and systemic health indicators to generate individualized risk profiles and predict therapeutic outcomes; this application is proposed here, as the cited studies concern diagnostic imaging rather than selection for retinal cell therapy [13,14,15].

Natural language processing (NLP) algorithms applied to electronic health records could automatically identify patients who meet eligibility criteria for cell therapy trials, flagging relevant diagnoses, medication histories, and prior surgical procedures that might affect transplantation outcomes [51]. Prognostic models trained on longitudinal imaging data from natural history cohorts could predict the rate of disease progression for individual patients, enabling more precise timing of intervention, intervening early enough to preserve residual photoreceptors that can integrate with transplanted cells, but not so early as to subject patients unnecessarily to invasive treatment [14]. Genetic stratification using AI-assisted variant classification may improve the accuracy of diagnosis and the identification of candidates for mutation-specific therapies.

Within this proposed framework, patients would be stratified into three illustrative risk tiers based on multimodal imaging-derived profiles and predicted likelihood of post-operative engraftment (Figure 2). The tiers are hypothetical, and no validated criteria or thresholds currently exist for assigning candidates for retinal cell therapy to such tiers. Tier 1 (favorable profile) reflects limited structural damage and high predicted regenerative potential; Tier 2 (intermediate profile) reflects moderate structural compromise; and Tier 3 (high-risk profile) corresponds to advanced disease with reduced predicted therapeutic response. This stratification would determine downstream clinical and manufacturing pathways. Tier 1 patients would proceed with standard manufacturing and post-operative protocols (Section 5.5), Tier 2 patients would be assigned enhanced monitoring and adapted treatment timelines, and Tier 3 patients would undergo individualized manufacturing optimization and intensified perioperative management, with consideration of alternative therapeutic strategies. Accordingly, Stage 1 would establish the foundational decision layer that would govern both manufacturing prioritization (Section 5.3) and longitudinal monitoring intensity (Section 5.5).

### 5.2. Stage 2: Gene Editing Planning and Safety Assessment

Following patient selection, AI tools guide the design of patient-specific CRISPR editing strategies. For each patient’s disease-causing genetic variant, AI-powered gRNA design platforms assess a full spectrum of candidate guide RNAs based on predicted on-target efficiency, off-target landscape, and chromatin accessibility at the target locus in the relevant iPSC line [80,81]. In parallel, genome-wide off-target prediction models estimate the likelihood of unintended genomic interactions, generating risk scores that inform candidate prioritization. Candidate guides are selected based on a combined assessment of predicted editing efficiency and safety, while those exceeding predefined risk thresholds are excluded from further consideration.

The selected editing strategy is modeled in silico to anticipate potential genotoxic risks, and the results inform the design of comprehensive safety testing panels for edited clones [53,80]. In the proposed framework, this would include automated whole-genome sequencing analysis pipelines integrating AI-based structural variant calling, copy number variation detection, and single nucleotide variant classification to provide a safety dossier for each edited clone. This is a proposed application: the cited tool [53] performs in silico variant-aware off-target detection and was not evaluated on sequenced edited iPSC clones.

ML models trained on prior editing datasets could predict the likelihood that a given clone harbors cryptic off-target events not detectable by standard bioinformatic methods, flagging clones for additional orthogonal validation [53,81]. In the proposed framework, such AI-assisted safety workflows could reduce the time and cost of clone characterization and improve confidence in clone safety; this has not been demonstrated in clinical-grade iPSC editing.

In cases where no candidate guide RNA satisfies combined efficacy and safety criteria, the workflow transitions to a non-edited cell replacement strategy, ensuring that therapeutic progression remains feasible while maintaining safety constraints. Accordingly, Stage 2 defines a critical decision point that determines whether downstream manufacturing, surgical, and monitoring workflows follow an edited or non-edited therapeutic pathway (Section 5.3, Section 5.4 and Section 5.5).

### 5.3. Stage 3: Cell Production, Differentiation, and Quality Control

Selected and characterized patient-derived or allogeneic iPSC clones are expanded and subjected to directed differentiation protocols under AI-monitored manufacturing conditions. Automated imaging platforms capture morphological data at defined timepoints, which are analyzed by AI models to predict differentiation efficiency and cell purity in near-real time. Deviating cultures are flagged for human review, enabling early corrective action [78]. At the end of differentiation, AI-powered flow cytometry analysis and single-cell transcriptomics provide comprehensive characterization of the cellular product, including cell type composition, purity, viability, and functional markers.

Release testing of manufactured cell products typically involves a battery of assays assessing identity, purity, potency, and safety. AI can accelerate and augment this process by identifying correlations between in-process quality indicators and final product attributes, potentially enabling earlier and more reliable prediction of product quality, reducing the time between manufacturing and clinical use [78,79]. Anomaly detection algorithms continuously monitor for out-of-specification parameters that might indicate contamination, equipment failure, or raw material deviations, enabling immediate corrective action and documentation for regulatory compliance [86].

Collectively, Stage 3 establishes an adaptive manufacturing environment in which AI-driven monitoring and predictive modeling enhance consistency, safety, and efficiency of therapeutic cell production prior to transplantation.

### 5.4. Stage 4: Surgical Delivery and Intraoperative Guidance

The surgical delivery of cell therapies to the retina is a technically demanding procedure requiring exceptional precision to deposit cells in the correct anatomical location without causing collateral damage to the delicate retinal architecture. Conventional surgical approaches rely on the skill of the operating surgeon, guided by real-time microscopy and occasionally intraoperative OCT [11,45]. This dependence can introduce inter-surgeon variability that may influence graft placement accuracy and clinical outcomes. AI-powered surgical assistance systems have been proposed as a way of integrating pre-operative imaging, real-time intraoperative guidance, and predictive analytics to support consistent and precise cell delivery; current supporting evidence is preclinical (Section 4.7).

In the proposed framework (see also Section 4.7), such systems would use patient-specific anatomical maps generated during pre-operative assessment to optimize surgical planning and guide subretinal injection trajectories. The patient’s immune profile, genetic background (when gene-edited cells are used), and disease stage, characterized during earlier stages of the therapeutic pipeline, could, in principle, inform perioperative planning. The specific examples of anesthesia strategy, surgical approach, and patient positioning for optimal retinal access are illustrative only and are not supported by direct evidence cited in this review. During the procedure, real-time feedback mechanisms and intraoperative imaging can verify graft localization and identify potential complications, enabling immediate corrective action when necessary. The resulting surgical and imaging data are subsequently integrated into the longitudinal monitoring framework, where post-operative multimodal imaging and AI-driven outcome prediction models assess graft survival, integration, and functional recovery over time (Section 5.5). Collectively, Stage 4 links pre-operative planning with longitudinal outcome evaluation, ensuring continuity across the therapeutic pipeline.

### 5.5. Stage 5: Post-Transplantation Monitoring and Outcome Prediction

Longitudinal monitoring following cell transplantation is essential for assessing graft survival and integration, detecting adverse events such as immune rejection or proliferative vitreoretinopathy, and measuring functional outcomes. AI-powered multimodal retinal image analysis can support sensitive and objective tracking of these parameters over time [11,14,15]. Automated segmentation of OCT images at multiple post-operative timepoints can quantify changes in retinal layer thicknesses, RPE integrity, and photoreceptor outer segment morphology that may reflect the status of the graft and host tissue; the cited studies were performed in non-transplanted retinal disease or gene-therapy cohorts, not in recipients of retinal cell grafts [72,73,74]. To systematically capture these structural and functional changes, the proposed framework envisages an illustrative longitudinal follow-up schedule that integrates multimodal imaging with functional assessments throughout the first post-transplant year (Figure 1 and Figure 2).

As an illustrative example, post-operative monitoring could follow a structured 12-month schedule with evaluations at Weeks 1, 4, 12, 24, and 52. These time points are proposed and are not derived from a validated protocol; follow-up schedules differ among published early-phase retinal cell-therapy trials [5,6,11,29]. As illustrated in Figure 1 and Figure 2, these evaluations include multimodal structural imaging combined with functional testing as described previously.

Monitoring intensity would be adapted according to the patient-specific risk stratification established during Stage 1. Low-risk patients (Tier 1) would undergo standard assessments at Weeks 1, 4, 12, 24, and 52. Intermediate-risk patients (Tier 2) would receive additional evaluations at Weeks 2 and 8, while high-risk patients (Tier 3) would undergo intensified surveillance at Weeks 1, 2, 4, 6, 8, 12, 24, and 52. This personalized monitoring strategy is hypothesized to improve early detection of complications while optimizing resource utilization; this hypothesis has not been tested.

Beyond quantitative image analysis, the proposed framework envisages translating longitudinal monitoring data into three illustrative decision points that could inform patient management and therapeutic adaptation; these are hypothetical and are not validated clinical decision rules.

Week 4: Early Integration Decision: Initial AI-generated predictions assess graft engagement, structural integration, and early host response. Patients demonstrating favorable trajectories would continue with standard post-operative management and immunosuppressive regimens. Conversely, indicators of poor graft integration, early inflammatory activity, or elevated rejection risk would prompt intensified monitoring and clinician consideration of escalating immunosuppressive or anti-inflammatory therapy before irreversible damage occurs; no validated AI-based thresholds for such decisions currently exist.

Month 3: Functional Assessment and Re-intervention Eligibility: Combined structural markers (OCT, FAF) and functional testing (visual acuity, kinetic perimetry, light sensitivity) would be used to estimate whether the patient is on trajectory for meaningful recovery. Unfavorable predictors could prompt discussion of re-intervention (for example, a repeat graft at Months 4–6 in selected high-risk candidates) or alternative therapies; the feasibility, safety, and timing of repeat grafting are unestablished, and this option is given as a hypothetical example only.

Month 12: Long-term Trajectory: Comprehensive evaluation of structural stability and functional recovery establishes the long-term therapeutic outcome. Patients with sustained success may be considered for treatment of the contralateral eye or enrollment in protocol extension studies. Individuals achieving stable but limited improvement would receive tailored visual rehabilitation plans and realistic long-term expectations. Cases of treatment failure would undergo detailed analysis to distinguish between graft integration failure, immunological rejection, disease progression, or surgical limitations.

Patient-centered communication would be integrated throughout the monitoring process. Outcome reviews would be conducted (illustratively) at Months 1, 3, 6, and 12, during which AI-generated visualizations and probability distributions would be used to explain graft status, expected recovery trajectories, and associated uncertainties. These tools facilitate shared decision-making regarding additional interventions, continuation of therapy, participation in follow-up studies, or transition to alternative treatment options.

Early detection of immune infiltration, graft contraction, abnormal vascular responses, or other adverse biological signatures may enable timely modification of immunosuppressive regimens and clinical management strategies, potentially preventing graft failure in patients where intervention remains feasible [12]. By shifting clinical practice from reactive management to predictive intervention, AI-enabled outcome forecasting could represent an important advance in regenerative ophthalmology if validated prospectively.

In the proposed framework, importantly, Stage 5 would complete the closed-loop regenerative medicine pipeline by feeding longitudinal outcome data back into earlier stages of the therapeutic process. Patterns of graft survival, immune rejection, functional recovery, and adverse events are incorporated into future patient stratification models (Stage 1), gene-editing optimization strategies (Stage 2), manufacturing quality-control algorithms (Stage 3, and AI-assisted surgical planning systems (Stage 4). This continuous learning framework is intended to enable iterative refinement of biological products, clinical protocols, and decision-support systems, progressively improving safety, efficacy, and personalization across successive patient cohorts. In this manner, post-transplantation monitoring serves not only as an assessment stage but also as a critical engine driving the ongoing evolution of the entire AI-enabled retinal regenerative medicine pipeline.

## 6. Discussion

### 6.1. Principal Findings and Synthesis

This review indicates that AI has shown meaningful, quantifiable performance in several application areas relevant to the cell-based retinal therapy pipeline, from patient stratification through post-transplantation monitoring, although most of the supporting evidence is retinal-adjacent, cross-domain, preclinical, retrospective, or conceptual rather than derived from recipients of retinal cell therapy. The evidence is not uniformly mature across domains, however, and this heterogeneity is itself an important finding. The most translational-ready applications are in retinal imaging and patient stratification [13,14,15], where AI tools have transitioned from proof-of-concept to regulatory-adjacent deployment (their use for selecting patients for retinal cell therapy remains proposed), and in CRISPR off-target safety assessment [52,80,81], where AI-guided gRNA design and off-target analysis tools have improved the prediction and detection of off-target activity, a computational benefit that has not itself been shown to improve the safety of edited iPSC clones. Applications in bioreactor manufacturing [86,91] and post-transplantation monitoring [11,70] are at an earlier stage, with evidence largely derived from non-retinal or retinal-adjacent settings. iPSC quality control [56,78] and retinal organoid characterization [64,66] remain largely at the in vitro validation stage, with independent clinical-scale validation yet to be reported.

A central interpretive point concerns the nature of AI’s contribution throughout this pipeline: in every domain reviewed, AI functions as an amplifier of existing biological and clinical processes rather than an autonomous decision-maker. The reported 40–70% increase in experimentally verified off-target sites detected [53] reflects improved detection sensitivity rather than a reduction in the biological occurrence of off-target editing, and improved detection does not eliminate the need for empirical safety testing. The 92–95% diagnostic accuracies of DL systems [13,14] reflect the ability to aggregate subtle image features at high speed and consistency, not the replacement of ophthalmological expertise in treatment planning. This distinction is critical for setting realistic expectations and for designing appropriate regulatory frameworks.

### 6.2. AI as Potential Infrastructure

We hypothesize that AI may become useful supporting infrastructure for scalable, reproducible cell-based retinal therapy. The evidence reviewed here supports plausible utility, but not demonstrated necessity; conventional, non-AI approaches may achieve comparable control at some pipeline steps. The complexity of a fully autologous pipeline, reprogramming, CRISPR correction, directed differentiation, quality release, surgical delivery, and post-operative monitoring, involves dozens of sequential process steps, each with independent sources of variability. Robust monitoring, prediction, and adaptive control at each node, whether AI-assisted or conventional, will be needed to limit error propagation through this pipeline, which is a fundamental barrier to reproducible clinical outcomes [10,11]; AI may offer scalable means of achieving this. This framing is a forward-looking hypothesis and should be distinguished from demonstrated benefit. We therefore distinguish (i) demonstrated benefit, largely in retinal imaging; (ii) plausible future utility, which applies to most manufacturing, gene-editing, surgical, and monitoring applications; and (iii) the authors’ hypothesis that AI may become supporting infrastructure at scale. This distinction has implications for how cell therapy developers, regulators, and funders should prioritize the validation of AI integration.

### 6.3. Gaps, Contradictions, and Limitations of the Evidence

Not all findings in the reviewed literature were concordant. Reported AI-based iPSC quality metrics vary substantially in both magnitude and type across studies [56,78], e.g., 95.8% pixel-wise segmentation accuracy versus 0.76–0.86 AUC for outcome prediction (metrics for different tasks that are not directly comparable), a discrepancy likely attributable to differences in training data composition, iPSC line diversity, imaging platforms, and ground truth annotation protocols, highlighting the critical need for standardized benchmarking datasets. Reported improvements in CRISPR off-target prediction and detection are not directly comparable, because they concern different endpoints (prediction of off-target activity versus detection sensitivity for experimentally verified sites) and different datasets, and none of the cited studies measured a reduction in the biological occurrence of off-target editing [52,53]. These discrepancies are not merely technical; they reflect a deeper absence of methodological standardization across the field that will need to be resolved before AI-based safety tools can be incorporated into regulatory submissions.

The majority of AI applications reviewed in manufacturing, differentiation optimization, and surgical guidance were demonstrated in vitro, in animal models, or in small-scale proof-of-concept settings; prospective human clinical evidence is conspicuously sparse and constitutes the most significant gap in the current literature. The absence of prospective trials is not merely a limitation of this review; it reflects the genuine early stage of the field and underscores the urgency of designing clinical studies with AI-based endpoints built in from the outset rather than added retrospectively.

### 6.4. Limitations of the Review

Several limitations of this review merit explicit acknowledgment. As a narrative comprehensive review, formal pooling of effect estimates was not performed, and selection of included studies involved author judgment rather than exhaustive systematic enumeration, introducing the possibility of selection bias toward positive or high-profile findings. Publication bias in the primary literature may have inflated reported AI performance metrics, as negative and null results are systematically underreported in this domain. The primary search was restricted to English-language publications from 2015 to 2025, with foundational papers identified through systematic citation chaining (backward and forward reference tracking of key articles and seminal studies). This pragmatic decision was justified by the recency of AI applications in this field, but it was one that may have missed relevant earlier work. An update search was performed to identify more recent literature (final search date: 23 September 2026); some included sources are preprints or conference abstracts, which are identified as such and were not used as the sole basis for strong conclusions. The assignment of evidence levels (Section 4) involved author judgment, and the performance metrics in Table 2 are study-specific and not directly comparable across rows. The rapidly evolving nature of both AI methodology and clinical cell therapy development means that some findings reported here may already be superseded by unpublished or in-press work at the time of reading.

## 7. Future Directions

### 7.1. Personalized and Precision Cell Therapy

The convergence of multi-omics profiling, federated learning, and foundation models represents the frontier of truly personalized cell therapy. Federated learning frameworks, which train AI models across distributed institutional datasets without centralizing patient-level data, offer a pathway to generalizable, privacy-preserving predictive systems particularly valuable for rare retinal dystrophies where no single center holds sufficient data to train robust models independently. Multi-omics integration combining genomic, transcriptomic, proteomic, metabolomic, and imaging data at the single-cell level may provide unprecedented resolution of individual biological states, potentially enabling improved prediction of treatment response, graft compatibility, and immunological risk compared with current clinical assessments. These foundation models pre-trained on massive biomedical datasets and fine-tuned on retinal disease-specific data may achieve improved diagnostic and prognostic accuracy, although this remains to be validated. Large language models integrated with structured clinical data may enable natural language interfaces for clinical decision support, making AI-powered insights accessible to clinicians without specialized data science expertise. Ultimately, AI-driven treatment matching, selecting the optimal cell product formulation, delivery route, and adjunctive immunosuppressive regimen for each patient’s genetic and phenotypic profile, may transform cell-based retinal therapy from a largely uniform intervention to a genuinely individualized one.

### 7.2. Next-Generation Organoid Models and AI-Accelerated Drug Discovery

Next-generation retinal organoid models incorporating vascularization, immune cell populations, and physical scaffold support are approaching greater fidelity to in vivo retinal architecture and physiology [31,32]. AI-powered analysis of these complex multi-component systems may enable more predictive preclinical modeling of cell therapy outcomes, including assessment of graft-host interactions, immune-mediated rejection dynamics, and long-term photoreceptor integration, that current two-dimensional culture models cannot provide. Additionally, high-throughput AI-assisted drug screening in patient-derived organoid disease models may accelerate identification of neuroprotective compounds, anti-inflammatory agents, and differentiation enhancers that can be combined with cell therapy to improve outcomes [84,85]. The development of AI-enabled organ-on-a-chip platforms incorporating retinal organoids interfaced with microfluidic vasculature and immune cell compartments may create physiologically relevant test beds for preclinical evaluation of combined cell–gene–drug therapeutic strategies, generating rich multimodal data streams amenable to iterative AI-driven model refinement; this is a prospective vision rather than an evidenced application.

### 7.3. Closed-Loop and Real-Time AI Guidance

Looking further ahead, the integration of AI into implantable retinal monitoring devices and smart drug delivery systems may enable closed-loop therapeutic approaches that continuously monitor retinal structure and function, adaptively modulating interventions in response to detected changes [45]. Such systems could detect early signs of graft rejection and trigger localized immunosuppressive release, or identify optimal electrical stimulation parameters for retinal prostheses matched to the surviving retinal circuitry of individual patients, applications that blur the boundary between therapy delivery and outcome monitoring [12,45]. Thus, intraoperative AI guidance systems may evolve from passive advisors to active co-operators in surgical workflows, dynamically adjusting delivery parameters, recommending target injection sites based on real-time imaging, and alerting surgeons to developing complications before they become irreversible [11,45]. Achieving this will require not only technical development but deliberate regulatory and ethical framework development governing autonomous and semi-autonomous AI systems in surgical contexts, ensuring appropriate human oversight and accountability at every stage.

### 7.4. Regulatory Science and Clinical Translation

Responsible clinical translation of AI-enhanced cell therapy workflows requires parallel development of regulatory science. Prospective clinical trials with pre-specified AI-based endpoints, transparent model cards documenting training data provenance and known failure modes, and post-market surveillance frameworks for continuously learning AI systems are all necessary components of a trustworthy regulatory pathway [92,93]. The FDA’s framework for AI/ML-based SaMD and the EMA’s reflection paper on AI in the medicinal product lifecycle [50] provide important starting points, but sector-specific guidance addressing AI embedded within cell therapy manufacturing processes and intraoperative surgical systems, where AI outputs directly determine patient safety, will be essential and has not yet been established, although existing medical-device, software, medicinal-product, combination-product, GMP, and quality-system frameworks may nevertheless apply [92,93].

### 7.5. Equity and Global Access

The equitable distribution of AI-enhanced cell therapies deserves explicit and proactive attention. Current AI models are predominantly trained on imaging and genomic data from high-income, European-ancestry populations, limiting performance and applicability in lower-income and genetically diverse settings [13,14]. As retinal diseases such as diabetic retinopathy and RVO disproportionately affect populations in low- and middle-income countries [19,21], the risk is that AI-enhanced cell therapies compound rather than reduce existing health disparities. Intentional diversification of training datasets, open-source model sharing, and health technology assessment frameworks accounting for global disease burden are essential prerequisites for ensuring that the benefits of this emerging field extend beyond well-resourced academic medical centers.

## 8. Conclusions

Cell-based therapies represent the most promising path toward true vision restoration in retinal degenerative diseases, yet their clinical translation has consistently been constrained by the complexity and variability of every stage of the therapeutic pipeline. This review has argued that AI may have the potential to become supportive infrastructure for this pipeline, although its necessity has not been demonstrated, with potential roles in stratifying patients, certifying iPSC quality, mapping CRISPR off-target landscapes, optimizing bioreactor processes, guiding surgical delivery, and detecting graft failure earlier. This potential nevertheless remains incompletely validated: most of the evidence reviewed is retinal-adjacent, cross-domain, preclinical, retrospective, or conceptual, and prospective evidence in retinal cell therapy is largely lacking.

What AI cannot do is equally important to state clearly. No algorithm restores a photoreceptor already lost. No model replaces the biological insight of a stem cell scientist or the judgment of a vitreoretinal surgeon. AI enhances but does not replace the biological, surgical, and clinical expertise that underpins cell-based retinal therapy, and this distinction must remain central to how the field develops, validates, and deploys these tools.

Three commitments will determine whether this convergence delivers on its promise: prospective clinical trials designed with AI-based endpoints from the outset; deliberate diversification of training datasets to reflect the global burden of retinal disease; and regulatory science that evolves in step with AI embedded within manufacturing and surgical systems. If sustained, the interdisciplinary collaboration of ophthalmologists, stem cell biologists, bioengineers, data scientists, and regulators may help bring durable, vision-restoring cell therapies for AMD, RP, Stargardt disease, and related conditions from aspiration to achievable clinical reality.

## Figures and Tables

**Table 1:** Number of clinical trials in ClinicalTrials.gov registered from 1 January 2015 to 31 December 2025 (11 calendar years; see note below).

Condition/Disease	Intervention/Treatment	Number of Studies
Retinal disease	Cell therapy	56
	iPSC	9
	CRISPR Gene Editing	2
	Artificial Intelligence	70

Note: The ClinicalTrials.gov searches were run on 31 December 2025 and were restricted to trials registered between 1 January 2015 and 31 December 2025. Searches were performed separately for each intervention/treatment term under the condition “retinal disease”, using the following exact search terms and filters: Cell therapy, iPSC, CRISPR Gene Editing, Artificial Intelligence. The categories are not mutually exclusive: a trial may be counted in more than one row, so the counts represent overlapping search results rather than unique trials. The count for “Artificial Intelligence” reflects trials retrieved by that search term and does not indicate that these trials involve AI-enhanced retinal cell therapy.

**Table 2:** (**A**) AI applications in patient selection and stratification, gene editing planning and safety assessment, and cell production, differentiation, and quality control (pipeline Stages 1–3). Evidence Level follows the classification in Section 4: Direct (studies using retinal cell products or transplanted retinal cells), Retinal-adjacent (retinal imaging, disease, organoid, or gene-therapy evidence without a cell-therapy product), Cross-domain (generic iPSC, non-retinal, or other proof-of-concept evidence), or Conceptual/preprint (non-peer-reviewed or in silico only). The “Study setting, sample size and validation” column states the experimental setting, sample size where reported, external-validation status, and whether retinal cell therapy was directly involved; “not reported” indicates that the item was not found in the cited abstract or article, and “n.a.” that it does not apply. Metrics are study-specific and not directly comparable across rows. (**B**) AI applications in surgical delivery and intraoperative guidance and post-transplantation monitoring and outcome prediction (pipeline Stages 4–5). Evidence Level classification, the “Study setting, sample size and validation” column, and comparability caveats are as described for Table 2A. (**C**) AI applications in retinal imaging and disease diagnosis. These studies are cross-cutting with respect to the five pipeline stages (informing patient selection, Section 5.1, and post-transplantation monitoring, Section 5.5) and, unless otherwise noted, were conducted in patients with retinal disease rather than in recipients of retinal cell therapy; they are therefore retinal-adjacent evidence rather than direct cell therapy evidence. The “Study setting, sample size and validation” column is as described for Table 2A.

(**A**)							
**Pipeline Domain**	**AI Architecture/Technique**	**Disease/Cell Type**	**Key Metric**	**Primary Outcome**	**Evidence Level**	**Study Setting, Sample Size and Validation**	**Ref.**
Patient stratification	NLP/ML on Electronic Health Record (EHR) data (narrative review)	Retinal diseases (all types)	Eligibility identification	Automated patient cohort identification	Cross-domain (review)	Narrative review of NLP in medicine and ophthalmology; no primary cohort. Sample size: n.a. External validation: n.a. Retinal cell therapy: no.	[51]
Patient stratification	Deep learning (bimodal fundus photography + OCT)	Multiple retinal diseases (incl. diabetic retinopathy, dry/wet AMD)	Diagnostic performance and cross-device generalizability	Multi-disease detection from bimodal imaging, supporting imaging-based patient assessment; stratification for cell therapy not demonstrated	Retinal-adjacent	Cross-sectional imaging study, 1445 fundus–OCT pairs from 1029 patients (three hospitals); additionally tested on datasets from other devices/scan patterns (88, 91 and 60 pairs); independent-site validation not stated. Retinal cell therapy: no.	[47]
Patient stratification	AI methods for diagnosis and progression forecasting (narrative review)	Glaucoma	Diagnosis and progression forecasting	Review of AI-based progression forecasting; no prognostic model for RP, AMD or Stargardt disease and no cell-therapy stratification demonstrated	Retinal-adjacent	Narrative review; sample size n.a.; external validation n.a. Retinal cell therapy: no.	[46]
CRISPR gene editing safety	Stacked ML regression models (Elevation)	Generic CRISPR-Cas9 off-target datasets (not iPSC- or retinal-specific)	Off-target activity prediction (guide–target and guide-summary scores)	Reported to outperform competing off-target scoring approaches on independent datasets (computational benchmark)	Cross-domain	In silico benchmark on published off-target datasets; sample size dataset-specific. Evaluated on independent datasets. Retinal cell therapy: no.	[52]
CRISPR gene editing safety	Variant-aware off-target search + ML activity scoring (VARSCOT)	Human genomic variation (1000 Genomes individuals); no sequenced edited clones	Detection sensitivity for experimentally verified off-targets	40–70% more experimentally verified off-targets detected than other software tools (detection sensitivity, not reduced off-target editing)	Cross-domain	In silico; 100 gRNAs compared across individual genomes and the reference; ML scoring model tested on an independent test dataset. Retinal cell therapy: no.	[53]
iPSC quality control	CNN (VGG13)	Human embryonic stem cell (hESC) colonies, line H9	Classification of good vs. bad colony phenotype	89% accuracy	Cross-domain	In vitro; single hESC line, 269 phase-contrast images, validation set *n* = 54; no external validation. Retinal cell therapy: no.	[54]
iPSC quality control	WND-CHARM supervised ML classifier (feature-based)	Human iPSC lines: bona fide iPSCs (1H) vs. improperly reprogrammed non-iPSCs (15B2)	Classification accuracy by cross-validation	Almost 100% accuracy discriminating bona fide iPSCs from non-iPSCs using immunofluorescence images of PML and Cajal nuclear bodies; genomic stability and tumorigenicity not assessed	Cross-domain	In vitro; 60 colony images for each of nine cell lines (540 images); cross-validation, no external validation stated. Retinal cell therapy: no.	[55]
iPSC quality control	U-Net (CNN) segmentation with automated colony-picking algorithm	Patient-derived hiPSC colonies (fibroblasts from 15 patients with inherited retinal degeneration)	Colony segmentation accuracy/IoU/Dice	95.8% pixel-wise accuracy, IoU 0.83; mean Dice 0.84 (34 images) and 0.81 (10 images from five unseen cell lines); automates colony picking, not a quality or pluripotency prediction	Cross-domain	In vitro. Phase-contrast images from 44 wells; model developed on ten patient lines (34 images) and tested on 10 images from five cell lines not used in training. Retinal cell therapy: no (reprogramming/picking automation only).	[56]
iPSC quality control	Multi-source feature ensemble (3 deep CNNs + 4 traditional descriptors)	iPSC colonies	3-class colony quality (good/medium/bad)	95.55% classification accuracy; quality labels verified by live-cell fluorescent staining; genomic or chromosomal stability not assessed	Cross-domain	In vitro; 46,500 colony images. External validation: not reported. Retinal cell therapy: no.	[57]
iPSC quality control	Graph attention autoencoder for unsupervised clustering (scVGATAE)	scRNA-seq datasets from several tissues and species (not iPSC-specific)	Clustering accuracy (ARI, NMI)	Highest mean ARI and NMI across nine datasets versus six methods (e.g., Goolam: +10.1% NMI, +23.2% ARI over scGAC); detection of aberrant iPSC subpopulations not demonstrated	Cross-domain	In silico; nine public scRNA-seq datasets (dozens to 7324 cells; up to 19 cell types) with database-provided labels; no experimental or external validation. Retinal cell therapy: no.	[58]
iPSC quality control	Reference-profile deconvolution (CellMap)	Primary and iPSC-derived cell types (neural, cardiac, hepatic and others)	Composition estimation (RMSE)	Median RMSE <0.2 for purified primary cell types (except pericytes) and <0.3 for iPSC-derived samples (except a subset of astrocytes); accuracy lower when true fraction <5%; tumorigenic or off-target subpopulations not specifically detected	Cross-domain	In silico; 17 datasets (nine major cell types) plus 9 CNS datasets; tested on primary and iPSC-derived samples and on pseudo-bulk mixtures from three training datasets; compared with MuSiC, SCDC and Bisque. Retinal cell therapy: no.	[59]
iPSC quality control	Heterogeneous graph transformer on single-cell multi-omics (MarsGT)	Simulated and real human multi-omic datasets; mouse retina application	Rare-population identification	Outperformed existing tools in identifying rare cells; in mouse retina data, revealed rare bipolar-cell and Müller glia subpopulations; not applied to iPSC-derived cultures or tumorigenicity	Retinal-adjacent	In silico; 550 simulated and four real human datasets, plus mouse retina data as an application; no experimental or external validation stated. Retinal cell therapy: no.	[60]
Differentiation optimization	Reinforcement learning (RL)	iPSC organoid (computational; preprint)	Final cell purity	69% win rate vs. random perturbation baseline (in silico only; not RPE purity)	Conceptual (preprint)	In silico only (preprint); iPSC-derived organoid scRNA-seq data; no experimental validation; external validation: not performed. Retinal cell therapy: no.	[61]
Differentiation optimization	CNN (ResNet50, DenseNet) on transmitted-light images	Mouse ESC to epiblast-like cells; human iPSC mesoderm differentiation	Early detection of differentiation onset	Distinguishes pluripotent from early-differentiating cells: >99% accuracy in mouse ESC, detectable ~20 min after induction; independent validation accuracy 0.996 (ResNet50) and 0.998 (DenseNet); in a second mouse ESC line, ~85% at 1 h, rising to >99% by 8 h; not RPE or final-purity prediction	Cross-domain	In vitro; ~800 training, 200 test and 50 final-validation images per group (later ~1000 per group); independent validation in three further mouse ESC experiments (1116 images); also tested in a second mouse ESC line and in human iPSC mesoderm differentiation. Retinal cell therapy: no.	[62]
Retinal organoid characterization	Two-photon imaging pipeline with custom StarDist3D deep-learning nuclei segmentation	Gastruloids derived from four mouse ESC lines (not retinal)	3D segmentation and quantification of nuclear morphology and gene expression	Nuclei segmentation F1 85 ± 3% at 50% IoU; quantified 3D gradients of gene expression and nuclear morphology; a morphometry tool, not a predictive model, and not applied to iPSC-derived or retinal cultures	Cross-domain	In vitro imaging study (reviewed preprint); no single total of gastruloids reported, n per analysis: 6 (PBS) and 9 (glycerol) for clearing, 37 for dual-view registration, 8 small + 5 large for expression gradients; 4414 manually annotated nuclei for training. Segmentation validated on manual annotations of the authors’ own samples; other datasets assessed qualitatively only. Retinal cell therapy: no.	[63]
Retinal organoid characterization	Deep learning on time-lapse imaging of organoids	Retinal organoids (RPE and lens tissue outcomes)	Early prediction of RPE and lens emergence and size, organoid by organoid	Accurately predicts emergence and size of RPE and lens tissue at early stages, before they are visually discernible; not validated in human iPSC-derived retinal organoids	Retinal-adjacent	In vitro (preprint); about 1000 organoids and >100,000 time-lapse images; external validation not stated. Retinal cell therapy: no.	[64]
Retinal organoid characterization	Variational autoencoders (vanilla, β-VAE, MMD-VAE) with latent space arithmetic	A549 lung cancer cells: Cell Painting morphology profiles and L1000 gene expression (not retinal or iPSC)	Reconstruction error and L2 distance between predicted and real polypharmacology profiles	MMD-VAE best at predicting polypharmacology cell states versus PCA and shuffled controls, but only for some mechanism combinations (most were close to shuffled controls); no retinal or iPSC application	Cross-domain	In silico, public data: 1571 compounds across six doses (10,368 Cell Painting consensus profiles; 50,303 replicate profiles) and 118,050 L1000 profiles; 80/10/10 train/validation/test split; arithmetic evaluated on all data including training; leave-five-out retraining as robustness check; no independent external dataset. Retinal cell therapy: no.	[65]
Drug screening	U-Net CNN with organoid-identification and tracking modules (OrganoID)	Pancreatic cancer organoids (training); lung, colon, adenoid cystic carcinoma and mouse intestinal organoids (testing); not retinal	Segmentation overlap (IOU), count and area agreement, tracking accuracy	Mean IOU 0.74 (PDAC) and 0.79 (other human organoids); count CCC 0.95 and area CCC 0.97; tracking accuracy >89% over 92 h; morphology changes in a gemcitabine dose-response; retinal compound screening not demonstrated	Cross-domain	In vitro imaging; trained on 52 and validated on 14 manually segmented PDAC images; tested on 28 human test images (PDAC plus 6 each of lung, colon, ACC), 19 mouse intestinal images and one 92 h time-lapse; small image sets, no independent laboratory validation; 3 mouse images failed the benchmark. Retinal cell therapy: no.	[66]
Cell manufacturing & bioreactor optimization	Hybrid model: LSTM or feedforward neural network coupled with material-balance equations	HEK293 fed-batch cultures, proprietary line (not iPSC or retinal)	Prediction of 27 extracellular state variables (WMSE, R^2^)	Best LSTM hybrid: R^2^ = 0.93 on test data; test WMSE about 13% lower than the best feedforward hybrid and less sensitive to data resampling, but more parameters and about 50% more CPU time; not applied to cell-therapy manufacturing	Cross-domain	In vitro culture data modelled in silico: 20 fed-batch cultures (Ambr 250 mL, 27 variables, no viral infection); 12 training, 4 validation and 4 test experiments in 10 random experiment-wise partitions; 784 model structures compared; no independent external dataset. Retinal cell therapy: no.	[67]
(**B**)							
**Pipeline Domain**	**AI Architecture/Technique**	**Disease/Cell Type**	**Key Metric**	**Primary Outcome**	**Evidence Level**	**Study Setting, Sample Size and Validation**	**Ref.**
Surgical delivery & intraoperative guidance	Computer vision + robot-assisted system	Subretinal injection (rabbit model; no human cell transplantation)	Placement accuracy	91.7% (robot-assisted) vs. 37.5% (manual) first-attempt success rate	Retinal-adjacent (preclinical)	Randomized preclinical study, 48 pigmented rabbits (24 per group). No independent replication. Retinal cell therapy: no (injection technique only).	[45,68]
Post-transplantation monitoring	Gradient-boosting ML (LightGBM) phase-specific models	Deceased-donor kidney transplant recipients (graft failure)	ROC AUC for graft failure in five post-transplant intervals	Test ROC AUC 0.92 (9–15 months), 0.84 (15–39), 0.73 and 0.72 (0–3 and 3–9), 0.70 (39–72); a solid-organ analogy only, not shown for RPE grafts	Cross-domain	Retrospective UNOS/OPTN registry data, 2015–2020/21; *N* = 298–828 per interval; 5-fold cross-validation with a 10% hold-out test set; a blinded external validation cohort is mentioned but its size is not reported; ethnic minority groups excluded. No RPE or photoreceptor graft data. Retinal cell therapy: no.	[69]
Post-transplantation monitoring	Modified U-Net deep-learning layer segmentation of SD-OCT	RPE65-associated inherited retinal degeneration before and after voretigene neparvovec gene therapy	Outer retinal layer distances and EZ area	Quantified outer-retina differences, including treatment-related effects; EZ diameter and area better preserved in paediatric eyes treated with gene therapy and in milder phenotypes; gene therapy, not cell therapy	Retinal-adjacent	Imaging study of 22 patients and 94 healthy controls; small dataset; no external validation stated. Retinal cell therapy: no (gene augmentation).	[70]
Post-transplantation monitoring	CNN patch classification of OCT B-scans with Otsu post-processing	Choroideremia and retinitis pigmentosa (inherited retinal degeneration)	Jaccard similarity with manual grading of preserved ellipsoid zone	Jaccard 0.894 (RP) and 0.912 (choroideremia) for preserved photoreceptor areas; no graft-related endpoints	Retinal-adjacent	Imaging study at one centre; 20 choroideremia and 22 RP subjects; trained on 10 eyes per disease, tested on 10 choroideremia, 12 RP and 5 healthy eyes; one OCT device; no external validation. Retinal cell therapy: no.	[71]
Post-transplantation monitoring	Hybrid U-Net and sliding-window CNN segmentation with manual correction	RPGR-associated X-linked retinitis pigmentosa	Dice score and Bland–Altman agreement for EZ area and outer-segment volume	Dice 0.8524 versus manual grading, comparable to 0.8417 between two graders; EZ area correlation 0.9928; the model alone, without manual correction, did not reach this agreement; no graft-related endpoints	Retinal-adjacent	Imaging study; 96 volume scans from 48 patients as test set; model trained on 280–960 mid-line B-scans from separate patients; one OCT device; no external validation. Retinal cell therapy: no.	[72]
(**C**)							
**Pipeline Domain**	**AI Architecture/Technique**	**Disease/Cell Type**	**Key Metric**	**Primary Outcome**	**Evidence Level**	**Study Setting, Sample Size and Validation**	**Ref.**
Retinal imaging & diagnosis	Pre-trained CNNs (VGG16, EfficientNetB0) with transfer learning	Diabetic retinopathy (colour fundus photographs)	Classification accuracy	VGG16 95.21% (Kaggle 2015 set) and EfficientNetB0 97.54% (APTOS 2019 set); different datasets and class definitions, so not directly comparable	Retinal-adjacent	Retrospective public datasets: Kaggle DR 2015 (35,126 images) and APTOS 2019 (3662 images); 70/15/15 train/validation/test split; no independent external validation. Retinal cell therapy: no.	[73]
Retinal imaging & diagnosis	CNN + (segmentation + classification net)	Retinal disease (general)	Diagnostic accuracy	94% referral accuracy across 50+ conditions	Retinal-adjacent	Retrospective; 997 test patients; additionally tested on a second OCT device type. Retinal cell therapy: no.	[14]
Retinal imaging & diagnosis	DL autosegmentation	AMD/geographic atrophy	Segmentation & progression	Predicts GA lesion area and annualized growth rate from 3D OCT	Retinal-adjacent	Retrospective analysis of lampalizumab GA-trial OCT; 1219 development/442 hold-out eyes. Hold-out from the same trials; no independent external cohort. Retinal cell therapy: no.	[74]

CNN = convolutional neural network, DL = deep learning, iPSC = induced pluripotent stem cell, LSTM = long short-term memory, ML = machine learning, RPE = retinal pigment epithelium, VAE = variational autoencoder, OCT = optical coherence tomography, RP = retinitis pigmentosa, AMD = age-related macular degeneration, AUC = area under the curve, ESC = embryonic stem cell, GA = geographic atrophy, HEK293 = human embryonic kidney 293 cells.

## Data Availability

No new data were created or analyzed in this study. Data sharing is not applicable to this article.
